# Cost-effectiveness Analysis of the Oncomine™ Dx Target Test MultiCDx System Using Next-generation Sequencing and Single-gene Test in Advanced and Recurrent Nonsquamous Non-small-cell Lung Cancer

**DOI:** 10.31662/jmaj.2023-0206

**Published:** 2024-07-10

**Authors:** Tomomi Kohara, Shunya Ikeda, Koichi Benjamin Ishikawa

**Affiliations:** 1 Graduate School of Public Health, International University of Health and Welfare, Tokyo, Japan

**Keywords:** Cost-effectiveness analysis, Cost-utility analysis, Lung cancer, Gene test, Next-generation sequencing, Oncomine

## Abstract

**Introduction:**

To determine the appropriate treatment for patients with advanced/recurrent nonsquamous non‒small-cell lung cancer (NSCLC), a companion diagnostic was conducted to detect driver mutations through genetic testing. In Japan, Oncomine Dx Target Test (DxTT) using next-generation sequencing (NGS) that can comprehensively detect gene mutations or single-gene tests are conducted as companion diagnostics. Furthermore, cost-effectiveness analysis was conducted to compare the cost-effectiveness of Oncomine DxTT using NGS with that of single-gene test in Japan.

**Methods:**

The target population included patients with advanced/recurrent nonsquamous NSCLC. A model structure was constructed for the Oncomine DxTT strategy and three single-gene tests (i.e., epidermal growth factor receptor (EGFR) mutations and anaplastic lymphoma kinase (ALK)/c-ros oncogene 1 (ROS1) rearrangements) with reference to previous studies and the Clinical Practice Guidelines of Lung Cancer 2022 in Japan. The model structure assumed that genetic testing would be conducted and first-line treatment used the drug most recommended in the 2022 Japanese Lung Cancer Clinical Practice Guidelines, depending on the driver mutation,. Model inputs were obtained from the literature and price list in Japan, and cost-utility analysis was conducted.

**Results:**

For the Oncomine DxTT strategy, the expected incremental costs and effectiveness were estimated to be approximately JPY 172,361 (JPY 12,285,228 *vs.* JPY 12,112,867 for strategies A and B, respectively) and −0.51 quality-adjusted life-year (QALY) per patient (21.93 QALY *vs.* 22.44 QALY for strategies A and B). As a result, the costs increased but the effectiveness decreased. Therefore, the Oncomine DxTT strategy was dominated by the three single-gene tests. Sensitivity and scenario analyses revealed that the test success rate of Oncomine DxTT affected the results.

**Conclusions:**

The genetic test using Oncomine DxTT before the first-line treatment is not cost-effective compared with the three single-gene tests (EGFR/ALK/ROS1) for patients with advanced/recurrent nonsquamous NSCLC.

## Introduction

In Japan, the number of patients with newly diagnosed lung cancer is increasing annually. According to statistics, in 2022, approximately 126,000 patients (84,000 men and 42,000 women) were diagnosed with lung cancer in 2019 ^(1)^. Furthermore, the number of deaths is increasing. The site-specific mortality rate was the highest in 1998. In 2020, the number of deaths from lung cancer was approximately 75,000 (53,000 men and 22,000 women) ^(1)^, the number of cancer deaths by site, lung cancer is the highest in men and the second highest in women. Furthermore, the mortality rate per 100,000 population is the highest for lung cancer ^(1)^. Although the 5-year relative survival rate tended to increase, it was 34.9% (29.5% for men and 46.8% for women) from 2009 to 2011 ^(2)^, and the prognosis was not better than that of other cancers. In Japan, the 5-year survival rate for non-squamous NSCLC worsens as the stage progresses, reaching 8.1% at stage IV. Consequently, the prognosis of stage IV disease is very poor. Therefore, early detection of lung cancer and provision of appropriate treatment are imperative.

Drug therapy for lung cancer has dramatically changed with the discovery of driver mutations. In 2002, gefitinib was first launched in Japan; however, its efficacy could not be demonstrated in a phase 3 trial in which genetic mutations had not been discovered. The discovery of epidermal growth factor receptor (EGFR) gene mutations in 2004 accelerated the discovery of gene mutations and the development of corresponding tyrosine kinase inhibitors by demonstrating high efficacy in patients with EGFR gene mutations in the IPASS study ^(4)^. As of December 2022, 18 molecularly targeted drugs have been approved in Japan for eight gene mutations in lung cancer: epidermal growth factor receptor (EGFR), anaplastic lymphoma kinase (ALK), c-ros oncogene 1 (ROS1), v-Raf murine sarcoma viral oncogene homolog (BRAF), rearranged during transfection (RET), mesenchymal-epithelial transition factor (MET) exon 14 skipping, neurotrophic receptor tyrosine kinase (NTRK), and c-Ki-ras2 Kirsten rat sarcoma viral oncogene homolog (KRAS).

The Japanese Guideline for Diagnosis and Treatment of Lung Cancer 2022 ^(5)^ recommends that various genetic tests (EGFR/ALK/ROS1/BRAF/MET/RET/KRAS/NTRK) and programmed death-ligand 1 (PD-L1) tests should be conducted for stage IV NSCLC, and based on the results, the appropriate drug therapy is determined. For treatment with molecularly targeted drugs, performing companion diagnostics (CDx) corresponding to each drug to detect genetic mutations is crucial. Genetic tests is recommended to be performed at the same time without prioritization.

Single-gene tests have been employed as companion diagnostics. In June 2018, “Oncomine™ Dx Target Test MultiCDx System (Oncomine DxTT)” using next-generation sequencing (NGS) was listed as a companion diagnostic test. As of November 2022, Oncomine DxTT can support the detection of five driver genes (EGFR mutation, ALK fusion gene, ROS1 fusion gene, BRAF V 600 E mutation, and RET fusion gene) in NSCLC and the determination of indications for treatment with 11 molecularly targeted drugs ^(6)^. At present, a single-gene test or Oncomine DxTT is available as a companion diagnostic test. In the Guide for Gene Panel Testing Using Next-Generation Sequencing in Patients with Lung Cancer ^(7)^, Oncomine DxTT is recommended before the initiation of first-line treatment for patients with advanced/recurrent NSCLC as multiple biomarkers can be simultaneously searched.

However, according to the results of a domestic questionnaire survey, the actual implementation rate of Oncomine DxTT is only approximately half of that of all genetic tests ^(8), (9)^. Rare driver mutations can be detected using a comprehensive test; however, no studies have investigated the cost-effectiveness of these diagnostic tests in Japan. Although five cost-effectiveness articles, including NGS (e.g., Oncomine DxTT), had been reported overseas, the results are not applicable in Japan because the approved tests, therapeutic drugs, and medical care systems differ depending on the country and year. Furthermore, the incidence rate of driver mutations differs among races ^(10)^. Therefore, this study was designed to analyze the cost-effectiveness of using Oncomine DxTT in patients with advanced/recurrent nonsquamous NSCLC and compare it with that of single-gene tests.

## Materials and Methods

### Study population and model structure

Patients with advanced/recurrent nonsquamous NSCLC were included in this study. Those with squamous cell carcinoma were excluded as the frequency of driver gene mutations was lower in squamous than in nonsquamous cell carcinoma. Japanese patients aged ≥18 years with Eastern Cooperative Oncology Group Performance Status of 0-1 and no history of chemotherapy were investigated with reference to the patient background of clinical studies on molecularly targeted drugs used as the first-line treatment in patients with advanced/recurrent NSCLC.

The real-world data on genetic testing in lung cancer in Japan conducted using the combination of three single-gene tests (EGFR/ALK/ROS1) ^(11), (12)^ was utilized as comparative controls. A model structure was constructed for the following four testing strategies, with reference to previous studies and the Clinical Practice Guidelines of Lung Cancer 2022 in Japan:

Strategy A: Oncomine DxTT

Strategy B: Three single-gene tests (EGFR/ALK/ROS1)

Strategy C: One single-gene test (EGFR)

Strategy D: No genetic testing

**Figure 1.**
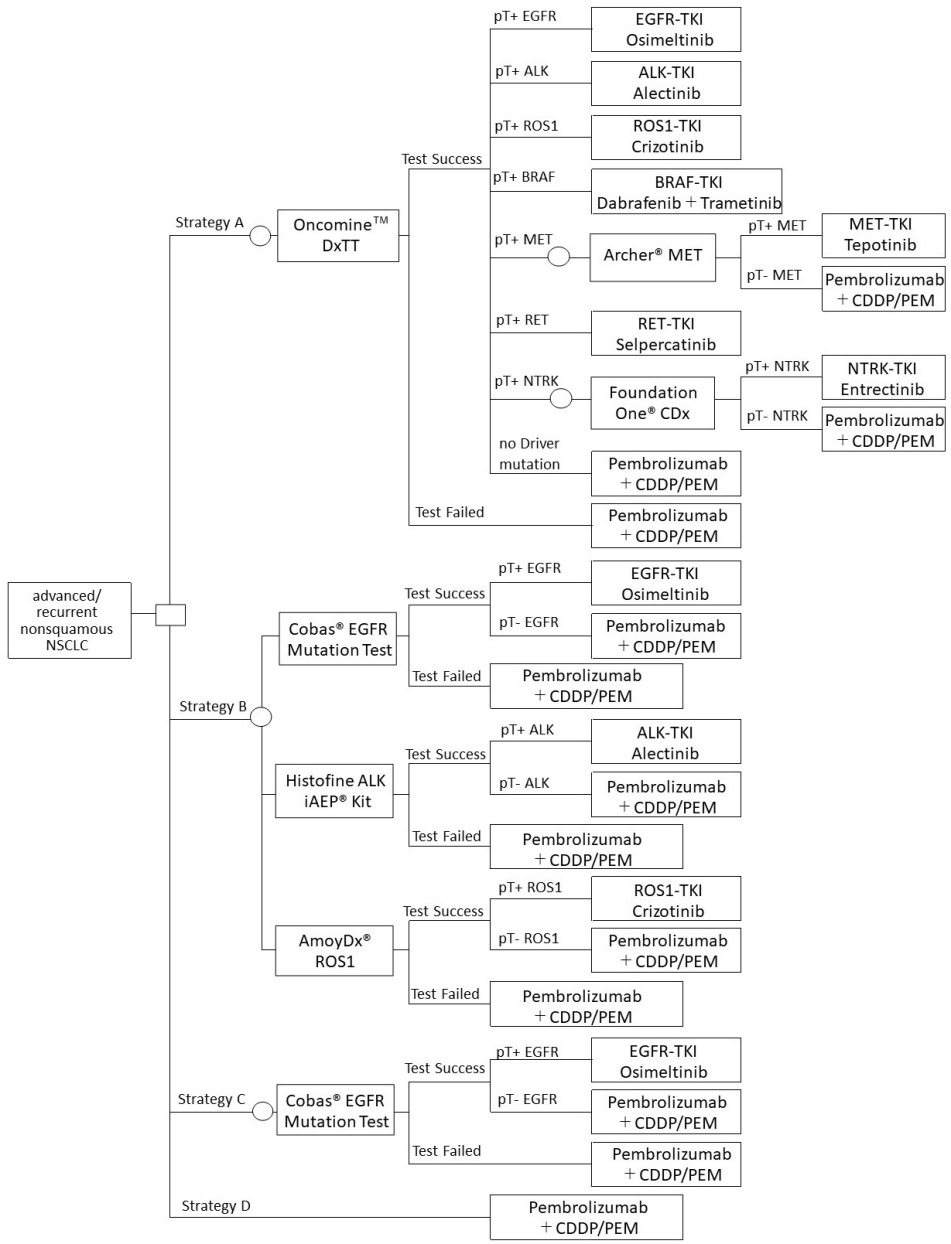
Model structure Strategy A: Oncomine DxTT Strategy B: Three single-gene tests (EGFR/ALK/ROS1) Strategy C: One single-gene test (EGFR) Strategy D: No genetic testing NSCLC, non-small cell lung cancer; PS, performance status; pT+, proportion of positive test (pTP + pFP); pT−, proportion of negative test (pTN + pFN); pTP, true-positive probability; pFP, false-positive probability; pTN, true-negative probability; pFN, false-negative probability; CDDP, cisplatin; PEM, pemetrexed.

The model structure shown in Figure 1 assumed that genetic testing would be conducted before the start of first-line treatment. Furthermore, it was assumed that the first-line treatment after detecting each driver gene mutation was the most recommended therapeutic drug based on the Clinical Practice Guideline of Lung Cancer 2022 in Japan. After the initiation of the first-line treatment, PD-L1 testing will not be considered, assuming that they will be conducted similarly in either strategy.

In case of failure in the test (unknown), it was assumed that no rebiopsy was performed and received the treatment of driver mutation negative. If the test was successful, the positive and negative rates were calculated as follows:

pT+: proportion of positive test (pTP + pFP)

pT−: proportion of negative test (pTN + pFN)

pTP: true-positive probability = Prevalence × Sensitivity × (1 − Unknown)

pFP: false-positive probability = (1 − Prevalence) × (1 − Specificity) × (1 − Unknown)

pTN: true-negative probability = (1 − Prevalence) × Specificity × (1 − Unknown)

pFN: false-negative probability = prevalence × (1 − Sensitivity) × (1 − Unknown)

Referring to the Guide for Gene Panel Testing Using Next-Generation Sequencing in Lung Cancer Patients ^(7)^, it was assumed that if METex 14 skipping mutation would be detected by Oncomine DxTT, the Archer^Ⓡ^ MET Companion Diagnostic System would be used. If the NTRK fusion gene would be detected, Foundation One^Ⓡ^ CDx would be used. For the single-gene test, a companion diagnostic test reimbursed for each drug would be used. Cobas^Ⓡ^ EGFR Mutation Test v2 was conducted, which has been approved as a companion diagnostic method for Osimertinib. The Histofine ALK iAEP^Ⓡ^ Kit for Alectinib and the AmoyDx^Ⓡ^ ROS1 Gene Fusions Detection Kit for Crizotinib were also used.

### Parameters

We assumed that the first-line therapy used was the most recommended molecularly targeted drug in the Clinical Practice Guidelines of Lung Cancer 2022 in Japan. Drugs with the same recommendation levels were defined as drugs that had been previously approved. As for the clinical outcome of each treatment, the progression-free survival (PFS) and overall survival (OS) were used with reference to clinical trials in the Clinical Practice Guidelines of Lung Cancer 2022 in Japan. If the results of the final analysis were published after the publication of the guidelines, they were used as reference, as appropriate. If Japanese or Asian data were available, they were prioritized.

The utility input was based on an international clinical trial involving patients with advanced/recurrent NSCLC (stage IIIB/IV) ^(13)^.

Because the proportion of EGFR mutations in the Japanese population is known to be higher than that in the America population, the prevalence was based on literature using Japanese database ^(10)^.

The costs were calculated from the direct costs, such as drug, test, disease monitoring, and outpatient chemotherapy costs, and the perspective of public medical care. Because the dose of each drug in overseas clinical trials referred to was the same as that in Japan, the dosage and administration were based on the package insert in Japan as of September 2022. The drug cost was calculated based on the 2022 National Health Insurance drug price list in Japan ^(14)^ with the assumption that payments had been made during the PFS period. All model parameters are shown in Table 1.

**Table 1.** Model Parameters.

	Base	Min	Max	Range	Reference
Prevalence					
EGFR	0.53	0.424	0.636	±20％	^(10)^
ALK	0.038	0.030	0.046	±20％
ROS1	0.009	0.007	0.011	±20％
BRAF	0.003	0.002	0.004	±20％
MET ex14 skipping	0.028	0.022	0.034	±20％
RET	0.019	0.015	0.023	±20％
NTRK	0.002	0.002	0.002	±20％
Other	0.371
Test success rate					
Oncomine™ DxTT	0.917	0.7336	1	±20％ (Max 1)	^(21)^
Cobas^Ⓡ^ EGFR Mutation Test v2	0.994	0.7952	1	±20％ (Max 1)
Histofine ALK iAEP^Ⓡ^ Kit	1	0.8	1	±20％ (Max 1)
AmoyDx^Ⓡ^ ROS1 Gene Fusions Detection Kit	1	0.8	1	±20％ (Max 1)
Archer^Ⓡ^ MET Companion Diagnostic System	0.9388	-	-	-	^(22)^
FoundationOne^Ⓡ^ CDx	1	-	-	-	^(23)^
Test performance					
Sensitivity Oncomine™ Dx TT	0.900		1.000	±20％ (Max 1)	^(24)^
Specificity Oncomine™ Dx TT	0.991		1.000	±20％ (Max 1)
Sensitivity Cobas^Ⓡ^ EGFR Mutation Test v2	0.808		1.000	±20％ (Max 1)	^(25)^
Specificity Cobas^Ⓡ^ EGFR Mutation Test v2	0.987		1.000	±20％ (Max 1)
Sensitivity Histofine ALK iAEP^Ⓡ^ Kit	0.980		1.000	±20％ (Max 1)	^(26)^
Specificity Histofine ALK iAEP^Ⓡ^ Kit	0.990		1.000	±20％ (Max 1)
Sensitivity AmoyDx^Ⓡ^ ROS1 Gene Fusions Detection Kit	1.00		1.000	±20％ (Max 1)	^(27)^
Specificity AmoyDx^Ⓡ^ ROS1 Gene Fusions Detection Kit	1.00		1.000	±20％ (Max 1)
Sensitivity Archer^Ⓡ^ MET Companion Diagnostic System	0.939	0.751	1.000	±20％ (Max 1)	^(22)^
Specificity Archer^Ⓡ^ MET Companion Diagnostic System	0.983	0.786	1.000	±20％ (Max 1)
Sensitivity FoundationOne^Ⓡ^ CDx	0.737	0.590	0.884	±20％	^(23)^
Specificity FoundationOne^Ⓡ^ CDx	1.000	0.800	1.000	±20％ (Max 1)
Utility					
First-line PF	0.71	0.67	0.76	95%CI	^(13)^
First-line PD	0.67	0.59	0.75	95%CI
Osimertinib（EGFR）					
mPFS	18.9	15.2	21.4	95% CI	^(28), (29)^
mOS	38.6	34.5	41.8	95% CI
Alectinib（ALK）					
mPFS	34.1	22.1	NR	95% CI	^(30)^
mOS	69.6	55.68	83.52	±20％
Crizotinib（ROS1）					
mPFS	15.9	12.9	24	95% CI	^(31)^
mOS	32.5	26	39	±20％
Dabrafenib + trametinib（BRAF）					
mPFS	10.9	7	16.6	95% CI	^(32)^
mOS	24.6	12.3	NE	95% CI
Tepotinib（MET）					
mPFS	11.0	1.4	NR	95% CI	^(33), (34)^
mOS	19.1	7.9	NR	95% CI
Selpercatinib（RET）					
mPFS	22	13.8	NR	95% CI	^(35)^
mOS	25.2	20.16	30.24	±20％
Entrectinib（NTRK）					
mPFS	14.9	11.9	17.9	±20％	^(36), (37)^
mOS	21	14.9	NE	95% CI
Pembrolizumab + chemotherapy					
mPFS	9.0	8.1	10.4	95% CI	^(38)^
mOS	22	19.5	24.5	95% CI
Drug cost (per month)					
Osimeltinib	¥621,582	¥497,266	¥745,898	±20％	^(14)^
Alectinib	¥808,452	¥646,762	¥970,142	±20％
Crizotinib	¥700,176	¥560,141	¥840,211	±20％
Dabrafenib + trametinib	¥1,761,432	¥1,409,146	¥2,113,718	±20％
Tepotinib	¥863,940	¥691,152	¥1,036,728	±20％
Selpercatinib	¥838,140	¥670,512	¥1,005,768	±20％
Entrectinib	¥906,570	¥725,256	¥1,087,884	±20％
Pembrolizumab + chemotherapy	¥910,163	¥728,130	¥1,092,195	±20％
Test cost（per one time)					
Oncomine™ Dx TT	¥140,000	¥112,000	¥168,000	±20％	^(6)^
EGFR/ALK/ROS1 (Cobas^Ⓡ^ EGFR, Histofine ALK, AmoyDx^Ⓡ^ ROS1)	¥60,000	¥48,000	¥72,000	±20％	^(39)^
EGFR/ALK	¥40,000	-	-	-
EGFR	¥25,000	-	-	-
Archer^Ⓡ^ MET Companion Diagnostic System	¥50,000	¥40,000	¥60,000	±20％	^(40)^
FoundationOne^Ⓡ^ CDx	¥50,000	¥40,000	¥60,000	±20％	^(41)^
Other cost					
Outpatient chemotherapy	-	-	-	-	^-^
Outpatient service fee	¥740	-	-	-	^(14), (42)^
Prescription fee	¥420	-	-	-
Prescription fee for anticancer drug	¥680	-	-	-
Outpatient chemotherapy	¥7,000	-	-	-
Intravenous drip fee (/day)	¥990	-	-	-
Preparation in sterile environment	¥450	-	-	-
Disease monitoring	-	-	-	-
Blood-drawing fee (/day)	¥370	-	-	-
Peripheral blood test fee	¥210	-	-	-
Peripheral blood test diagnostic fee	¥1,250	-	-	-
Biochemical test fee	¥1,060	-	-	-
Biochemical test diagnostic fee	¥1,450	-	-	-
Tumor marker test fee	¥4,080	-	-	-
CT scan with a contrast medium	¥10,200	-	-	-
CT scan diagnostic fee	¥4,500	-	-	-

### Cost-effectiveness analysis

Cost-utility analysis (CUA) with an effectiveness index of quality-adjusted life-year (QALY), which is often used in cost-effectiveness analysis in oncology, was conducted. QALY were calculated assuming that PFS was maintained at the utility of progression free (PF) and that the time from progression to death was maintained at the utility of progressive disease (PD). The median PFS and median OS were used because the average PFS and OS could not be calculated from previous studies. (QALY = mPFS × PF utility + (mOS − mPFS) × PD utility). The test success rate, test sensitivity/specificity, and incidence of driver mutations were used as input data to calculate the costs and QALY of each strategy.

The incremental cost-effectiveness ratio (ICER) between the strategies was calculated, and the strategy was judged to be cost-effective if the ICER was less than the threshold (JPY 7,500,000 in oncology) according to the Central Social Insurance Medical Council, an advisory body of the Ministry of Health, Labour and Welfare, which is authorized to determine the official prices paid to medical institutions from public health insurance, such as medical service fees and drug prices.

### Sensitivity analysis

Each parameter was varied at the upper and lower limits, and the parameters that had a strong influence on the results were evaluated. Those with 95% confidence intervals (CIs) in reference articles were preferentially used, whereas those without 95% CIs were changed at ±20%.

### Scenario analysis

Hypothetical scenarios were developed to determine whether they affected the results ^(15)^.

## Results

### Base case analysis

The results of the base case analysis are shown in Table 2 and 3. When Oncomine DxTT was conducted for patients with advanced/recurrent nonsquamous NSCLC before the first-line therapy, the expected incremental costs and effectiveness were estimated to be approximately JPY 215,064 (JPY 12,298,099 *vs.* JPY 12,083,034 for strategies A and B, respectively) and −0.16 QALY per patient (21.52 QALY *vs.* 21.68 QALY for strategies A and B, respectively) (Table 2).

**Table 2.** Base Case Results.

Strategy	Cost per patient (JPJPY)	Incremental cost (JPJPY)	Effectiveness (QALY)	Incremental effectiveness (QALY)	ICER (ΔCost/ΔQALY)
Strategy B: Three single-gene tests (EGFR/ALK/ROS1)	¥12,083,034	-	21.68	-	Dominant
Strategy A: Oncomine™ Dx TT	¥12,298,099	¥215,064	21.52	−0.16

**Table 3.** Base Case Results (Cost, Effectiveness of Each Strategy).

	Cost per patient (JPJPY)	Effectiveness (QALY)	ICER (ΔCost/ΔQALY)
Strategy D: No genetic testing	¥10,236,314	15.10	-
Strategy C: One single-gene test (EGFR)	¥11,172,603	20.07	¥188,281
Strategy B: Three single-gene tests (EGFR/ALK/ROS1)	¥12,083,034	21.68	¥566,433
Strategy A: Oncomine™ Dx TT	¥12,298,099	21.52	Dominated

As a result, the costs increased; however, the effectiveness decreased. Oncomine DxTT was dominated by the three single-gene tests.

The ICER of the one single-gene test (EGFR) strategy compared with the no-test strategy was calculated to be approximately JPY 188,281/QALY, indicating cost-effectiveness. The ICER of the three single-gene test (EGFR/ALK/ROS1) strategy compared with that of the one single-gene test (EGFR) strategy was calculated to be approximately JPY 566,433/QALY, indicating cost-effectiveness (Table 3).

A plot figure was created with QALY and cost. Oncomine DxTT is located in a region above the straight line formed by the three single-gene tests strategy and one single-gene test strategy. As a result, the Oncomine DxTT strategy was extremely dominated. This means that if the three single-gene and one single-gene test strategies are conducted at a certain proportion, the effects obtained with Oncomine DxTT are equivalent, and the cost is low, or the effects obtained at the same cost are high.

### Sensitivity analysis

Sensitivity analysis was conducted with 95% CI or ±20% as the lower and upper limits,　respectively. The ICERs were calculated when the success rate of Oncomine DxTT was the upper limit, the success rate of the Cobas EGFR test was the lower limit, the specificity of Oncomine DxTT was the lower limit, the specificity of ArcherMET was the lower limit, the specificity of FoundationOne was the lower limit, and the sensitivity of the Cobas EGFR test was the lower limit. In all cases, the results indicated that the Oncomine DxTT strategy was cost-effective compared with the three single-gene test strategy.

Otherwise, the results indicated that the Oncomine DxTT strategy was not cost-effective compared with the three single-gene test strategy (Table 4).

**Table 4.** Sensitivity Analysis Results.

					Min			Max		
Parameters	Base	Min	Max	Range	ΔQALY	ΔCost	ICER	ΔQALY	ΔCost	ICER
Prevalence: EGFR	0.53	0.424	0.636	±20％	−0.43	¥195,140	Dominated	−0.60	¥149,581	Dominated
Prevalence: ALK	0.038	0.0304	0.0456	±20％	−0.49	¥185,972	Dominated	−0.54	¥158,749	Dominated
Prevalence: ROS1	0.009	0.0072	0.0108	±20％	−0.51	¥172,633	Dominated	−0.51	¥172,088	Dominated
Prevalence: BRAF	0.003	0.0024	0.0036	±20％	−0.51	¥167,346	Dominated	−0.51	¥177,375	Dominated
Prevalence: MET ex14	0.028	0.0224	0.0336	±20％	−0.50	¥178,290	Dominated	−0.53	¥166,431	Dominated
Prevalence: RET	0.019	0.0152	0.0228	±20％	−0.52	¥142,014	Dominated	−0.50	¥202,707	Dominated
Prevalence: NTRK	0.002	0.0016	0.0024	±20％	−0.51	¥172,336	Dominated	−0.51	¥172,385	Dominated
Utility: First line Progression-free	0.71	0.67	0.76	95%CI	−0.51	¥172,361	Dominated	−0.52	¥172,361	Dominated
Utility: First line Progressive disease	0.67	0.59	0.75	95%CI	−0.46	¥172,361	Dominated	−0.56	¥172,361	Dominated
mPFS: Osimeltinib (EGFR)	18.9	15.2	21.4	95% CI	−0.51	¥269,850	Dominated	−0.52	¥106,490	Dominated
mOS: Osimeltinib (EGFR)	38.6	34.5	41.8	95% CI	−0.40	¥172,361	Dominated	−0.60	¥172,361	Dominated
mPFS: Alectinib (ALK)	34.1	22.1	NR	95% CI	−0.51	¥207,672	Dominated	−0.51	¥152,292	Dominated
mOS: Alectinib (ALK)	69.6	55.68	83.52	±20％	−0.48	¥172,361	Dominated	−0.55	¥172,361	Dominated
mPFS: Crizotinib (ROS1)	15.9	12.9	24	95% CI	−0.51	¥179,761	Dominated	−0.51	¥152,379	Dominated
mOS: Crizotinib (ROS1)	32.5	26	39	±20％	−0.50	¥172,361	Dominated	−0.53	¥172,361	Dominated
mPFS: Dabrafenib + trametinib (BRAF)	10.9	7	16.6	95% CI	−0.51	¥153,315	Dominated	−0.51	¥200,197	Dominated
mOS: Dabrafenib + trametinib (BRAF)	24.6	12.3	NE	95% CI	−0.53	¥172,361	Dominated	−0.50	¥172,361	Dominated
mPFS: Tepotinib (MET)	11	1.4	NR	95% CI	−0.53	¥−165,178	¥313,162	−0.51	¥249,713	Dominated
mOS: Tepotinib (MET)	19.1	7.9	NR	95% CI	−0.81	¥172,361	Dominated	−0.41	¥172,361	Dominated
mPFS: Selpercatinib (RET)	22	13.8	NR	95% CI	−0.52	¥49,330	Dominated	−0.51	¥238,377	Dominated
mOS: Selpercatinib (RET)	25.2	20.16	30.24	±20％	−0.57	¥172,361	Dominated	−0.45	¥172,361	Dominated
mPFS: Entrectinib (NTRK)	14.9	11.92	17.88	±20％	−0.51	¥168,581	Dominated	−0.51	¥176,140	Dominated
mOS: Entrectinib (NTRK)	21	14.9	NE	95% CI	−0.52	¥172,361	Dominated	−0.51	¥172,361	Dominated
mPFS: Pembrolizumab + chemotherapy	9.0	8.1	10.4	95% CI	−0.51	¥172,361	Dominated	−0.51	¥172,361	Dominated
mOS: Pembrolizumab + Chemotherapy	22	19.5	24.5	95% CI	−0.51	¥172,361	Dominated	−0.51	¥172,361	Dominated
Test Success Rate: Oncomine™ DxTT	0.917	0.7336	1	±20％ (Max 1)	−1.88	¥−209,422	¥111,509	0.11	¥345,141	¥3,265,726
Test Success Rate: Cobas^Ⓡ^ EGFR Mutation Test v2	0.994	0.7952	1	±20％ (Max 1)	0.69	¥392,501	¥569,742	−0.55	¥165,716	Dominated
Test Success Rate: Histofine ALK iAEP^Ⓡ^ Kit	1	0.8	1	±20％ (Max 1)	−0.26	¥312,298	Dominated	−0.51	¥172,361	Dominated
Test Success Rate: AmoyDx^Ⓡ^ ROS1 Gene Fusions Detection Kit	1	0.8	1	±20％ (Max 1)	−0.50	¥175,593	Dominated	−0.51	¥172,361	Dominated
Test Performance: Sensitivity Oncomine™ DxTT	0.900	0.72	1	±20％ (Max 1)	−2.89	¥−1,131,752	¥391,610	−0.36	¥247,184	Dominated
Test Performance: Specificity Oncomine™ DxTT	0.991	0.793	1	±20％ (Max 1)	20.77	¥15,328,4557	¥738,009	−0.51	¥172,361	Dominated
Test Performance: Sensitivity Archer^Ⓡ^ MET Companion Diagnostic System	0.939	0.75104	1	±20％ (Max 1)	−0.58	¥124,865	¥−216,707	−0.49	¥187,842	Dominated
Test Performance: Specificity Archer^Ⓡ^ MET Companion Diagnostic System	0.983	0.7864	1	±20％ (Max 1)	1.81	¥1,898,776	¥1,050,673	−0.71	¥23,077	Dominated
Test Performance: Sensitivity FoundationOne^Ⓡ^ CDx	0.737	0.5896	0.8844	±20％	−0.52	¥168,581	Dominated	−0.51	¥176,140	Dominated
Test Performance: Specificity FoundationOne^Ⓡ^ CDx	1.000	0.8	1	±20％ (Max 1)	2.17	¥2,731,206	¥1,257,468	−0.51	¥172,361	Dominated
Test Performance: Sensitivity Cobas^Ⓡ^ EGFR Mutation Test	0.808	0.6464	0.9696	±20％ (Max 1)	0.82	¥394,758	¥481,412	−1.15	¥35,730	Dominated
Test Performance: Specificity Cobas^Ⓡ^ EGFR Mutation Test	0.987	0.7896	1	±20％ (Max 1)	−1.24	¥17,749	Dominated	−0.46	¥182,221	Dominated
Test Performance: Sensitivity Histofine ALK iAEP^Ⓡ^ Kit	0.980	0.784	1	±20％ (Max 1)	0.08	¥352,203	¥4,402,538	−0.51	¥172,361	Dominated
Test Performance: Specificity Histofine ALK iAEP^Ⓡ^ Kit	0.990	0.792	1	±20％ (Max 1)	−6.43	¥−3,292,145	¥511,998	−0.51	¥172,361	Dominated
Test Performance: Sensitivity AmoyDx^Ⓡ^ ROS1 Gene	1.00	0.80	1	±20％ (Max 1)	−0.15	¥217,585	Dominated	−0.52	¥171,756	Dominated
Test Performance: Specificity AmoyDx^Ⓡ^ ROS1 Gene	1.00	0.80	1	±20％ (Max 1)	−1.61	¥−62,518	¥38,831	−0.49	¥176,524	Dominated
Drug Cost: Osimeltinib	¥621,582	¥497,266	¥745,898	±20％	−0.51	¥267,125	Dominated	−0.51	¥77,597	Dominated
Drug Cost: Alectinib	¥808,452	¥646,762	¥970,142	±20％	−0.51	¥191,672	Dominated	−0.51	¥153,049	Dominated
Drug Cost: Crizotinib	¥700,176	¥560,141	¥840,211	±20％	−0.51	¥179,865	Dominated	−0.51	¥164,856	Dominated
Drug Cost: Dabrafenib + trametinib	¥1,761,432	¥1,409,146	¥2,113,718	±20％	−0.51	¥161,903	Dominated	−0.51	¥182,819	Dominated
Drug Cost: Tepotinib	¥863,940	¥691,152	¥1,036,728	±20％	−0.51	¥97,746	Dominated	−0.51	¥246,975	Dominated
Drug Cost: Selpercatinib	¥838,140	¥670,512	¥1,005,768	±20％	−0.51	¥108,750	Dominated	−0.51	¥235,971	Dominated
Drug Cost: Entrectinib	¥906,570	¥725,256	¥1,087,884	±20％	−0.51	¥168,709	Dominated	−0.51	¥176,012	Dominated
Drug Cost: Pembrolizumab plus Chemotherapy	¥1,092,195	¥873,756	¥1,310,634	±20％	−0.51	¥183,704	Dominated	−0.51	¥161,017	Dominated
Test Cost: Oncomine™ DxTT	¥140,000	¥112,000	¥168,000	±20％	−0.51	¥144,361	Dominated	−0.51	¥200,361	Dominated
Test Cost: EGFR/ALK/ROS1	¥60,000	¥48,000	¥72,000	±20％	−0.51	¥184,361	Dominated	−0.51	¥160,361	Dominated
Test Cost: Archer^Ⓡ^ MET Companion Diagnostic System	¥50,000	¥40,000	¥60,000	±20％	−0.51	¥172,361	Dominated	−0.51	¥172,361	Dominated
Test Cost: FoundationOne^Ⓡ^ CDx	¥50,000	¥40,000	¥60,000	±20％	−0.51	¥172,361	Dominated	−0.51	¥172,361	Dominated
Other Cost: Tyrosine kinase inhibitors	¥31,700	¥25,360	¥38,040	±20％	−0.51	¥172,831	Dominated	−0.51	¥171,890	Dominated
Other Cost: Pembrolizumab + chemotherapy	¥45,173	¥36,138	¥54,208	±20％	−0.51	¥172,830	Dominated	−0.51	¥171,891	Dominated

### Scenario analysis

Scenario analysis was conducted based on some hypotheses. If the success rate of each strategy was 100%, the ICER was calculated to be approximately JPY 4,875,388/QALY. If the success rate of Cobas EGFR was comparable to that of Oncomine DxTT, the ICER was calculated to be approximately JPY 285,657/QALY. The values were lower than the threshold of JPY 7,500,000, indicating that the Oncomine DxTT strategy was cost-effective compared with the three single-gene test strategy. Other scenarios did not impact the results (Table 5).

**Table 5.** Scenario Analysis Results.

	Scenario	ΔQALY	ΔCost	ICER (ΔCost/ΔQALY)
Scenario 1	The prevalence of gene mutations is based on overseas data ^(15)^	0.220	¥831,845	JPY3,781,114/QALY
Scenario 2	Treatment of CDDP + PEM for negative driver gene mutation	0.530	¥−2,357,678	Dominant
Scenario 3	The effect of tepotinib (MET) is based on the results of all analyses (VISION study) instead of the Japanese subanalysis	−0.220	¥127,164	Dominated
Scenario 4	The effect of crizotinib (ROS1) is based on the results of all analyses (PROFILE1001) instead of Asian analysis	−0.080	¥231,504	Dominated
Scenario 5	The success rate for each genetic test is 100%	0.390	¥383,509	JPY983,356/QALY
Scenario 6	The sensitivity/specificity of each genetic test is 100%	−0.580	¥112,417	Dominated
Scenario 7	Archer MET, FoundationOne test is not conducted after Oncomine DxTT	−0.080	¥244,589	Dominated
Scenario 8	The test success rate of Cobas EGFR is comparable to that of Oncomine DxTT (91.7%)	0.220	¥285,657	JPY285,657/QALY
Scenario 9	The QALY of tepotinib (MET) and entrectinib (NTRK) is comparable to that of Crizotinib (ROS1) (QALY=22.4)	0.210	¥215,064	JPY1,024,114/QALY

## Discussion

This study was conducted to compare the cost-effectiveness between Oncomine DxTT and three single-gene tests for patients with advanced/recurrent nonsquamous NSCLC. A model structure was developed and used to analyze the effectiveness and cost of companion diagnostic tools in Japan. Furthermore, CUA was conducted. In conclusion, the Oncomine DxTT strategy was not cost-effective compared with the three single-gene test strategy.

Cost-effectiveness analyses comparing NGS and single-gene tests for NSCLC have been conducted in five foreign studies ^(16), (17), (18), (19), (20)^, and the results indicated that using a control strategy similar to the one used in this study was not cost-effective ^(16)^. Contrarily, four studies reported that NGS was cost-effective, although the strategies were slightly different ^(17), (18), (19), (20)^. One study reported that NGS-based parallel testing is cost-effective compared with single-gene-based sequential testing ^(17)^. Another study showed that multigene panel sequencing is known to have moderate cost-effectiveness compared with single-marker genetic testing ^(18)^. In addition, another study reported that upfront NGS represents a feasible, cost-effective method for diagnostic molecular profiling compared with sequential testing strategies ^(19)^. Finally, another study reported that NGS testing may provide a cost-effective strategy compared with combinations of single-gene tests ^(20)^. To the best of our knowledge, this is the first cost-effectiveness analysis to compare NGS with the single-gene test for NSCLC in Japan.

Although NGS is recommended in the Japanese NGS guidelines, we hypothesized that NGS would be cost-effective by detecting rare driver mutations and using molecularly targeted therapeutic drugs tailored to the driver mutations based on the guidelines and a previous study. However, the results were opposite to the expectations. The reason was considered to be the test success rate of Oncomine DxTT. When the test success rate of Oncomine DxTT exceeded 99.2%, the conclusion was that Oncomine DxTT was cost-effective compared with the three single-gene test strategy. Tepotinib (MET fusion gene) and Entrectinib (NTRK fusion gene) are newly covered by the national health insurance, and few studies have been reported. The referenced phase I/II study had a small number of patients. Therefore, we considered that the low QALY may have affected the results. However, the sensitivity and scenario analyses did not affect the results.

In this study, analysis was conducted using a simulated model, not data from a large database, by extrapolating the results of previous studies to a model, and some limitations were identified. The costs were calculated by stacking each cost, such as drug and test costs, while excluding hospitalization cost and management cost of adverse events, among others. Therefore, they may differ from the actual cost. The detection rate of driver mutations may differ from the actual rate as it was calculated based on the prevalence and test success rate in previous studies. For example, the detection rate of EGFR has been reported to be 25%-40% in Japan but approximately 50% in this study. For the prevalence, an article describing the prevalence of all target driver mutations in the Japanese population was used; however, this is the prevalence of lung adenocarcinoma, which is not exactly consistent with that of nonsquamous NSCLC. This is one of the study limitations. Furthermore, this cost-effectiveness analysis based on a hypothetical model structure with reference to the guidelines and testing as well as treatment flow may be different from the actual clinical practice because test and treatment are considered from various perspectives in the latter. To construct the model structure, several conditions were set, such as considering the first-line treatment but not the second-line or subsequent treatments, not considering the PD-L test and rebiopsy, and referring to overseas data if no Japanese data were available.

Further study in line with actual clinical practice using real-world data, such as diagnosis procedure combination data, electric health data, and health insurance claims data, is warranted. In addition, creating a detailed model structure based on actual tests and treatment flow is imperative.

This study was conducted on December 2022, and the treatment pattern and input data were different from those in December 2023, during which the manuscript was submitted. As of December 2023, Oncomine DxTT can support the detection of six driver genes (EGFR mutation, ALK fusion gene, ROS1 fusion gene, BRAF V 600 E mutation, RET fusion gene, and HER2 mutation) in NSCLC and the determination of indications for treatment with 14 molecularly targeted drugs. Trastuzumab deruxtecan has been approved for ERBB2 mutations, and sotorasib has been approved for the KRAS G12C mutation. The 2023 Japanese Guideline for Diagnosis and Treatment of Lung Cancer has been published, and some treatments and tests have changed from 2022. Analysis using the latest treatment models and input data is warranted.

In this study, we found that Oncomine DxTT was not cost-effective compared with the three single-gene tests (EGFR/ALK/ROS1). Although the turnaround time (TAT) was not considered in this study, the TAT in general is approximately 1-2 weeks for Oncomine DxTT and approximately 1 week for the single-gene test. Therefore, the test should be selected from various perspectives, including the cost-effectiveness and the condition of each patient. For these reasons, the results were considered to reflect the actual situation that the implementation rate of Oncomine DxTT was approximately half in Japan, although it is recommended in the guidelines.

Furthermore, based on the available input data, the single-gene test demonstrated higher true-positive rates for EGFR, ALK, and ROS1 compared with Oncomine DxTT. To replace Oncomine DxTT with a single-gene test, it is necessary to improve not only the test success rate but also accuracy. Moreover, within the current medical service system in Japan, there are issues regarding insurance coverage for tests. For instance, Oncomine DxTT after single-gene tests may not be covered by health insurance. Health insurance only covers tests conducted using companion diagnostics that are compatible with molecularly targeted therapeutic drugs. Considering that various therapeutic drugs will be launched in the future, establishing an environment that makes it easier to conduct genetic testing is necessary.

### Conclusion

In conclusion, this analysis revealed that the use of Oncomine DxTT in patients with advanced/recurrent nonsquamous NSCLC is not cost-effective in Japan compared with the three single-gene tests (EGFR/ALK/ROS1).

## Article Information

### Conflicts of Interest

Although the Tomomi Kohara is an employee of a pharmaceutical company, did not receive any financial support such as the school fee or this research expenses. This research does not include the product of the author’s company, and this company will not benefit or suffer any disadvantage from the results of this research.

### Acknowledgement

Author would like to express deep appreciation to Dr. Shunya Ikeda and Dr. Koichi Benjamin Ishikawa, who provided advices for this study, Dr. Yuji Tada, who provided advice on the study from a clinical perspective, and the public health specialists, classmates, and people in the laboratory who helped us conduct this study. And thank Enago (www.enago.jp) for the English language review.

### Author Contributions

TK and SI were primarily responsible for the study conception and design, analysis and interpretation of data, and drafting and revising the manuscript. KBI was responsible for reviewing the manuscript. All authors have approved the final version of this manuscript for publishing and agree to be held accountable for all aspects of the work.

### Approval by Institutional Review Board (IRB)

Not applicable.
